# The effects of different light qualities on the growth and nutritional components of *Pleurotus citrinopileatus*

**DOI:** 10.3389/fnut.2025.1554575

**Published:** 2025-04-28

**Authors:** Xiaoli Chen, Yihan Liu, Wenzhong Guo, Xiaoming Wei, Mingfei Wang, Xin Zhang, Wengang Zheng

**Affiliations:** ^1^Intelligent Equipment Research Center, Beijing Academy of Agriculture and Forestry Sciences, Beijing, China; ^2^College of Horticultural and Landscape Architecture, Tianjin Agricultural University, Tianjin, China

**Keywords:** *Pleurotus citrinopileatus*, LED light, free amino acid, volatile substances, flavor

## Abstract

Light is one of the key factors affecting the flavor of edible fungi. *Pleurotus citrinopileatus* were planted in a growth chamber in order to investigate the effects of different LED lights on the growth and development. Five treatments were set up in the experiment, namely white light (CK, as control), pure green light (G), pure blue light (B), pure red light (R) and far-red light (Fr). The results showed that: (1) R or Fr treatment caused deformities in *Pleurotus citrinopileatus*, showing a soft stipe, thin pileus, and shallow color. Compared with the control, the stipe length of *Pleurotus citrinopileatus* significantly decreased by 12.52% under treatment B, while the stipe diameter, pileus diameter, and fruiting body weight significantly increased by 35.52%, 18.30%, and 23.66%, respectively (*P* < 0.05). The color of *Pleurotus citrinopileatus* was more plump under B treatment, among which the spectral color parameters C and Hue increased by 2.72% and 1.64%, respectively. (2) B increased the proportion of umami and sweet amino acids [(UAA+SAA)/TAA] while decreased that of bitter amino acids in total amino acids (BAA/TAA) in *Pleurotus citrinopileatus* relative to the control. In addition, except for B treatment, other treatments (G, R, Fr) significantly reduced the content of mushroom flavored amino acids (e.g., Asp and Glu). (3) B increased the odor activity value (OAV) of key aroma compounds in *Pleurotus citrinopileatus* compared with the other light qualities in this study, while R increased the OAV of 1-octen-3-ol and 1-octen-3-one. However, considering that mushrooms cannot grow normally under R treatment, this study recommended blue light as the main light quality for industrial production of *Pleurotus citrinopileatus*.

## 1 Introduction

*Pleurotus citrinopileatus*, also known as golden top pleurotus, is a precious edible and medicinal fungus belonging to the family *Pleurotus* and genus *Pleurotus*. *Pleurotus citrinopileatus* is widely distributed in northeastern and southwestern regions of China (1). The fruiting body of *Pleurotus citrinopileatus* is yellow funnel-shaped, hence it is called the “flower of fungi”. *Pleurotus citrinopileatus* has extremely high nutritional value, rich in nutrients such as protein, polysaccharides, minerals, and essential amino acids for the human body. It also has various medicinal values such as antioxidant, blood pressure lowering, and cholesterol lowering (2). In recent years, it has been widely cultivated due to delicious taste and rich nutrition.

Flavor compounds are the main active components that contribute to the flavor of edible fungi, and are also important indicators for evaluating the taste and aroma of edible mushrooms. Free amino acids, also known as non protein amino acids, mainly affect the taste of edible mushrooms. At present, 15 types of free amino acids were studied in edible fungi, each with its unique taste, such as sour, sweet, bitter, and umami, and their content directly affects the freshness of mushrooms. Yamaguchi et al. (3) found that aspartic acid (Asp) and glutamic acid (Glu) were the major contributing components of mushroom umami taste. Other studies reported that the content of Asp and Glu in different mushrooms might vary between 0.05 and 45.85 mg/g (4, 5). Volatile substances such as alcohols, esters, ketones, aldehydes, alkanes and alkenes are the major contributing components of mushroom olfactory flavor. Alcohols, especially octacarbon alcohols, are characteristic substances commonly found in edible fungi. Studies have shown that the aroma of fresh mushrooms were mainly attributed to mushroom flavored substances such as 1-octen-3-ol, 3-octanol, 1-octanol, 1-octen-3-one, and 3-octanone (6–8). Ketones endow edible fungi with floral and fruity aromas, and the fragrance is long-lasting. Aldehydes have fruity and floral flavors and are key components of mushroom flavor compounds. Esters including ethyl hexanoate, ethyl octanoate and hexyl acetate give a very pleasant taste and greatly affect consumers' choices. Some mushrooms exhibit pleasant sweetness and mushroom flavor, while others exhibit bitterness and sourness due to these substances (9–12).

Light plays an important role in the synthesis and metabolism of flavor compounds which decide the flavor of green plants (13–15). For example, Colquhoun et al. (16) reported that red light increased the content of 3-methyl-1-butanal and 2-methyl-butanal in tomato fruits. Fan et al. (14) studied the effects of different spectra and daylight integrals on the volatile compounds of Micro Tom tomatoes, finding that high daylight integrals of green light and low daylight integrals of red light would reduce the content of volatile compounds in tomato fruits. Liu et al. (17) reported that white light was more conducive to the accumulation of flavor compounds such as total furan compounds, 2,6-dimethylpyrazine, and dibutyl sulfide in sweet melon compared with monochromatic light or the darkness. In addition to green plants, photo-sensitivity also exists in non-photosynthetic edible fungi. However, since edible fungi don't undergo photosynthesis, their light responses are different from those of green plants. Edible fungi regulate physiological responses in the body by sensing light signals through photoreceptors (18). Xsu et al. (19) found that pulsed ultraviolet light (PUV) could increase the vitamin D content in *Pleurotus citrinopileatus*. Hu et al. (20) found that light with a wavelength of 720 nm could increase the ergosterol content in *Pleurotus citrinopileatus*. Ye et al. (21) studied the effects of different light qualities on the hyphae and primordia differentiation of *Pleurotus ostreatus*, finding that more hyphae were formed and primordia differentiation was faster under blue light irradiation compared with red light irradiation or the darkess. Du et al.'s (22) study also has shown that blue light promoted the enlargement of mushroom pileus and pigment accumulation in *shiitake mushrooms*. Feng et al. (23, 24) found that blue light could enhance the aroma of dried *Suillus granulosus* compared to red, green, yellow, and white light. In addition, Yao et al. (25) reported that ultraviolet light could increase the content of tannin, total phenols, total flavonoids, and β–glucan as well as antioxidant properties in dried *L. edodes*. Wen et al. (26) studied the effects of different pulsed light on the volatile compounds of harvested *shiitake mushrooms* and found that pulsed light irradiation increased the content of 1-octen-3-ol, 1-octen-3-one, 3-octanol, and umami amino acids in *shiitake mushrooms*. Rathore et al. (27) found that UV-B irradiation could increase the content of amino acids, especially Glu, in dried *Calocybe indica*. It can be seen that light has a significant impact on the morphology, taste, and flavor of edible mushrooms.

Compared with traditional light sources, Light emitting diodes (LEDs) has become an important supplementary light source for facility edible mushroom production due to its unique advantages such as high photoelectric conversion, low thermal radiation, and adjustable light quality. At present, most reports on the light effects of mushrooms are based on dried mushrooms after harvesting, while there is relatively little study based on the growing mushrooms. In addition, there has been no study about the light effects on the growth and nutritional components of *Pleurotus citrinopileatus*. Therefore, this study evaluated the free amino acids and volatile substances of fresh *Pleurotus citrinopileatus* exposed to different LED light irradiation to screen the optimal light environment for *Pleurotus citrinopileatus* cultivation. This article aimed to provide a theoretical basis for light quality regulation in the industrial production of *Pleurotus citrinopileatus*.

## 2 Materials and methods

### 2.1 Experimental design

The study was conducted in a growth chamber at Beijing Academy of Agricultural and Forestry Sciences. An LED light system that could set any light formula was used as the experimental light. The *Pleurotus citrinopileatus* mushroom bags were treated with different light qualities from the day when the mycelium was full. Five treatments were set up in the experiment, namely white light (CK, as control), pure green light (G), pure blue light (B), pure red light (R) and far-red light (Fr), with a light/dark (L/D) period of 12 h/12 h for each treatment. The wavelength peak of green ligh, blue light, red light and far-red light were, respectively, 530 nm, 450 nm, 660 nm, and 735 nm. Each treatment had 25 *Pleurotus citrinopileatus* mushroom bags. The growth cycle of the mushrooms were divided into three stages that were button stage (rice shaped mushroom buds), young mushroom stage (round pileus) and mature mushroom stage (funnel-shaped pileus). The light intensity for each stage was 5 μmol·m^−2^·s^−1^, 15 μmol·m^−2^·s^−1^ and 30 μmol·m^−2^·s^−1^, respectively. The wavelength peak of green light, blue light, red light and far-red light were 520 nm, 450 nm, 660 nm, and 735 nm, respectively. The light intensity and spectrum were all measured approximately 10 cm below the light source using a spectrometer (LI-180, LI-COR, USA) (Table 1). The temperature, the CO_2_ concentration and the relative humidity of air in the growth chamber were 26 ± 1°C, 500 μmol·mol^−1^ and (90±1)%, respectively, during the entire growth period of *Pleurotus citrinopileatus*. Purified water was automatically sprayed twice a day at 8 am and 8 pm, for 1 min each time.

**Table 1:** LED light formula for different treatments of *Pleurotus citrinopileatus*.

**Treatment**		**Light supply mode**	**Light intensity (**μ**mol**·**m**^**−2**^·**s**^**−1**^**)**				
			**White light**	**Green light**	**Blue light**	**Red light**	**Far-red light**
Button stage	CK	White light	5	0	0	0	0
	G	Pure green light	0	5	0	0	0
	B	Pure blue light	0	0	5	0	0
	R	Pure red light	0	0	0	5	0
	Fr	Far-red light	0	0	0	0	5
Young mushroom stage	CK	White light	15	0	0	0	0
	G	Pure green light	0	15	0	0	0
	B	Pure blue light	0	0	15	0	0
	R	Pure red light	0	0	0	15	0
	Fr	Far-red light	0	0	0	0	15
Mature mushroom stage	CK	White light	30	0	0	0	0
	G	Pure green light	0	30	0	0	0
	B	Pure blue light	0	0	30	0	0
	R	Pure red light	0	0	0	30	0
	Fr	Far-red light	0	0	0	0	30

### 2.2 Sampling and phenotypic measurement

The stipe length, stipe diameter and pileus diameter of *Pleurotus citrinopileatus* were measured with a vernier caliper at the young mushroom stage and mature mushroom stage. The weight of *Pleurotus citrinopileatus* was measured by an electronic balance at mature mushroom stage. Eight fruiting bodies randomly taken from per treatment was regarded as a repetition, and there were three repetitions in each treatment.

### 2.3 Color parameter determination

The color parameters of *Pleurotus citrinopileatus* pileus were measured using a spectrophotometer (YS3010, Shenzhen San' enshi Technology Co., Ltd., Guangzhou, China). The spectral data was processed to obtain the color saturation (C), hue angle (Hue), color index (CCI) and color ratio (a^*^/b^*^). The larger the C, the higher the color saturation of the pileus. The Hue reflected the coloring of the pileus. CCI could be used to evaluate changes in the color of pileus. a^*^/b^*^ was the comprehensive color index. The calculation formula was as follows:


(1) $$\begin{array}{l} Hue=tan^{-l}\left(b^{*}/a^{*}\right) \end{array}$$

(2) $$\begin{array}{l} C=\sqrt{a^{*2}+b^{*2}} \end{array}$$

(3) $$\begin{array}{l} CCI=1000a^{*}/L^{*}b^{*} \end{array}$$

(4) $$\begin{array}{l} \text{Color ratio}={\text{a}}^{*}/b^{*} \end{array}$$

### 2.4 Free amino acids determination

The content of free amino acids was determined referring the method proposed by Boogers et al. (28). 0.5 g mushroom sample mixed with 20 mL pure water was extracted for 10 min in boiling water, and then diluted to 50 mL. Then, free amino acids standard solution, borate buffer, and derivatizing agent were added into a derivatization tube (6 mm × 50 mm) for derivatization. The mixture was placed at room temperature for 1 min, and then detected in an automatic amino acid analyzer (LA8080).

### 2.5 Volatile substance determination

#### 2.5.1 Sample extraction

Samples were extracted by solid-phase microextraction technology (SPME). 3.0 g mushroom freeze-dried sample and 2-methyl-3-heptanone (internal standard substance) were mixed in a 15 mL headspace bottle. Extraction head that had been aged at 250°C for 2 h was extracted at 35°C, and then desorpted at 250°C in the headspace bottle.

#### 2.5.2 GC-MS analysis

HP-5MS capillary column (30 m × 0.25 mm × 0.25 μm) was used for chromatographic separation, and Shimadzu GC-MS QP2010 gas chromatography-mass spectrometer was used for determination. Chromatographic conditions: the inlet temperature was 28°C, the carrier gas was helium (99.999%) with a flow rate of 3.0 mL/min, and the split ratio was 8:1. Temperature gradient program: the initial temperature of column during testing was maintain at 40°C for 1 min, the initial temperature increased to 160°C at 3°C /min, and increased to 230°C at 15°C /min, and held for 1 minute. Mass spectrometry conditions: EI ionization source, ionization voltage −70eV, ion source temperature 230°C, quadrupole temperature 150°C, solvent delay time 2 min. The data collection mode was full scan mode: 50–550 m/z.

### 2.6 Statistical analysis

SPSS Statistics 22 software was used for data processing and variance analysis, Origin 2021 and Hiplot software were used for plotting, and SIMCA 14.1 software was used for orthogonal partial least squares discrimination analysis (OPLS-DA). The data was represented as the mean ± standard deviation (SD).

## 3 Results

### 3.1 Phenotypic trait analysis of pleurotus citrinopileatus under different light quality

**Figure 1:**
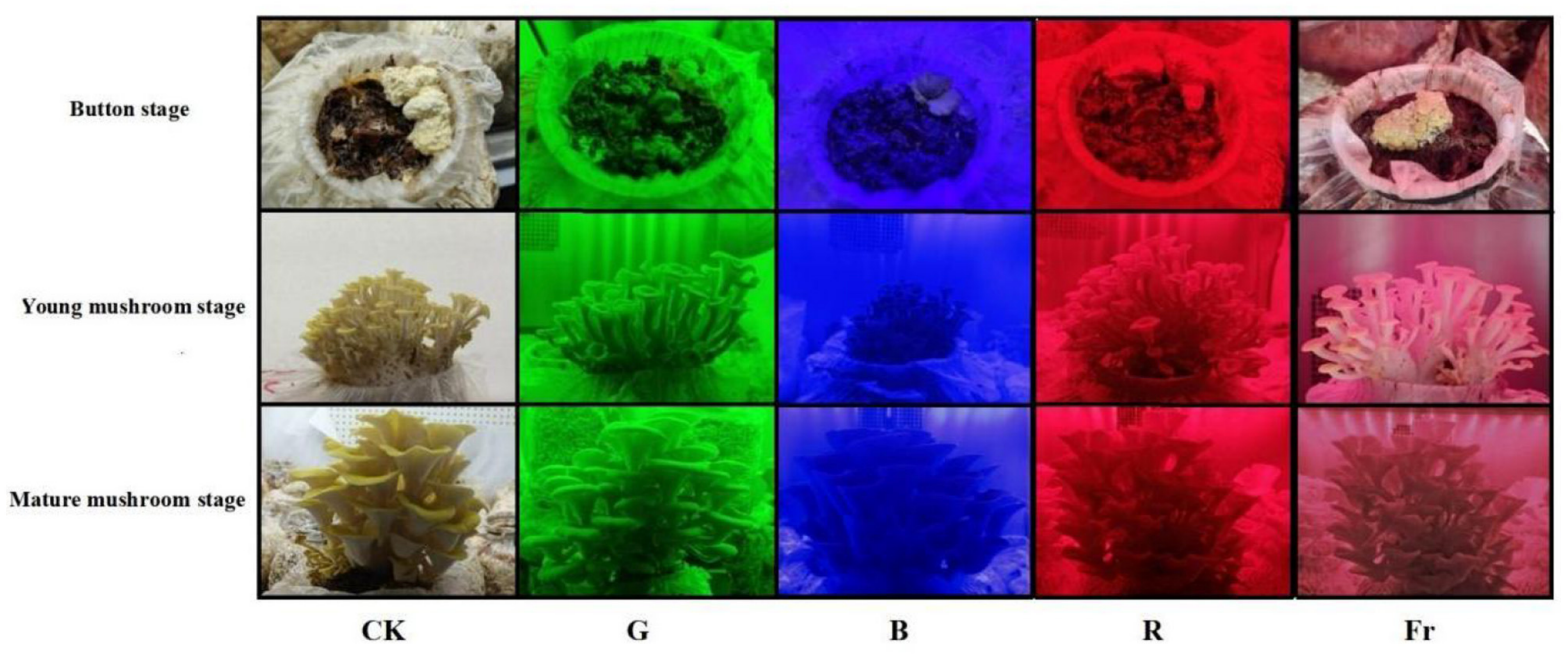
Photos of *Pleurotus citrinopileatus* at different growth stages under different light treatments.

As shown in Figure 1, photomorphogenic analysis revealed distinct phenotypic responses in *Pleurotus citrinopileatus* to specific light wavelengths. Compared with the white light (CK), the longer stipe and larger pileus of *Pleurotus citrinopileatus* fruiting body at all stages were found under green light irradiation. Mushroom fruiting bodies were the shortest and most compact under blue light treatment. The mushrooms exposed to red or far-red light grew normally during the button stage and young mushroom stage, but showed deformities at the mature mushroom stage, manifesting loose fruiting bodies, soft stipe, thin pileus, and light color.

**Figure 2:**
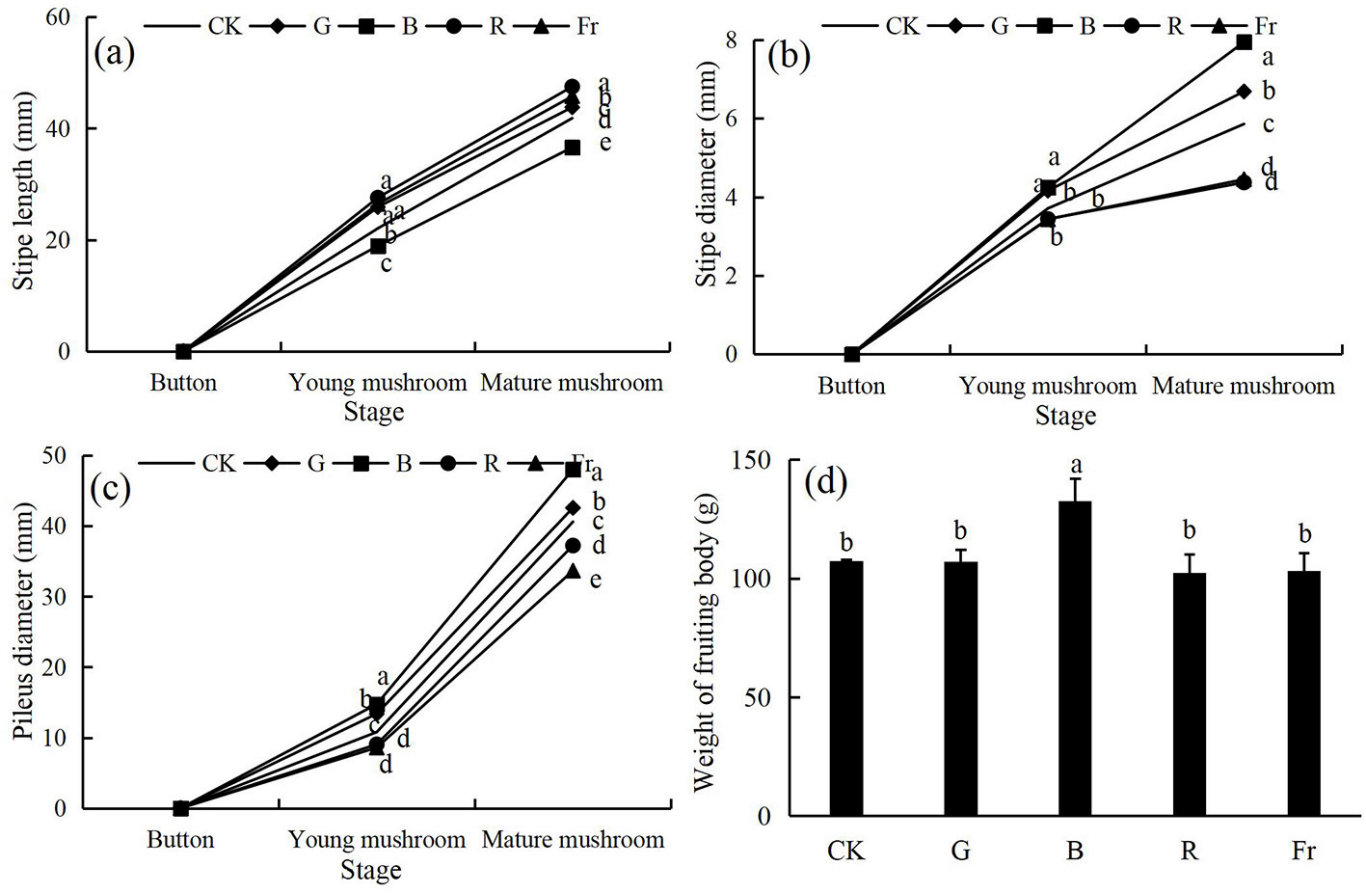
The stipe length **(a)**, stipe diameter **(b)**, pileus diameter **(c)** and the weight **(d)** of *Pleurotus citrinopileatus* under different light treatments. Different lowercase letters indicate significant differences between groups (*P* < 0.05).

As shown in Figure 2, compared with the control, the stipe length of *Pleurotus citrinopileatus* exposed to G treatment was significantly increased by 4.68%, while that exposed to B treatment was decreased by 12.52% (*P* < 0.05) (Figure 2a). The stipe diameter of mushroom exposed to G and B treatments were significantly increased by 14.11% and 35.52%, respectively (Figure 2b). The pileus diameter under these two treatments were significantly increased by 4.86% and 18.30%, respectively (*P* < 0.05) (Figure 2c). As shown in Figure 2d, the weight of fruiting bodies exposed to B treatment was significantly increased by 23.66% relative to the control (*P* < 0.05), while that under other treatments was decreased by 0.10%-4.45%.

### 3.2 Color spectral parameter of pleurotus citrinopileatus under different light quality

Considering that the color of *Pleurotus citrinopileatus* was mainly presented by the pileus, this study analyzed the color of the pileus of *Pleurotus citrinopileatus*. The C and Hue values of *Pleurotus citrinopileatus* pileus exposed to B treatment increased by 2.72% and 1.64% respectively, while the CCI and a^*^/b^*^ values decreased by 44.62% and 80.00% respectively, compared with the control. Meanwhile, the darkest simulated color of *Pleurotus citrinopileatus* was observed under the B treatment. On the contrary, the simulated color was lighter under R and Fr treatments, further indicating that monochromatic red light or far-red light was not conducive to the development of *Pleurotus citrinopileatus* (Table 2). The color difference of pileus might be due to the different light qualities perceived by mushroom photoreceptors, leading to different transcriptional reactions within cells (29), which indirectly affected the expression of pigment related genes or enzyme activities.

**Table 2:** Color spectral parameters of *Pleurotus citrinipileatus* pileus in each treatment.

**Treatment**	**C**	**Hue**	**CCI**	a^*^/b^*^	**Simulated color**
CK	59.84 ± 4.58b	87.01 ± 0.50a	0.65 ± 0.12b	0.05 ± 0.01b	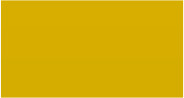
G	53.25 ± 6.70ab	84.51 ± 1.17b	1.21 ± 0.23a	0.10 ± 0.02a	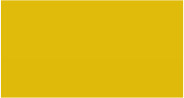
B	61.47 ± 4.54a	88.44 ± 1.34a	0.36 ± 0.32b	0.01 ± 0.02b	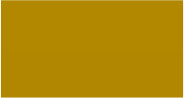
R	40.14 ± 9.28c	82.48 ± 1.97b	1.54 ± 0.40a	0.13 ± 0.04a	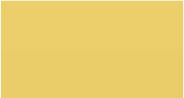
Fr	42.56 ± 7.90bc	84.19 ± 1.24b	1.28 ± 0.29a	0.10 ± 0.02a	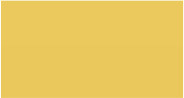

Different lowercase letters indicate significant differences of 0.05 in the color among treatments.

### 3.3 Free amino acids analysis of *Pleurotus citrinopileatus* under different light quality

As shown in Table 3, 15 free amino acids out of the 16 amino acids detected were identified in *Pleurotus citrinopileatus* subjected to all light treatments. Among which, the content of alanine (Ala) and leucine (Leu) was the highest (close to 97.12–146 mg/100 g), while that of methionine (Met) was the lowest (18.87–27.02 mg/100 g). However, arginine (Arg) was not identified in *Pleurotus citrinopileatus* irrespective of different light treatments.

**Table 3:** Free amino acid content of *Pleurotus citrinipileatus* under different LED light treatments.

**Amino acid**	**Amino acid content/(mg/100g)**				
	**CK**	**G**	**B**	**R**	**Fr**
Asp	76.44 ± 0.09a	49.16 ± 10.44c	62.32 ± 11.15bc	63.35 ± 1.29b	58.66 ± 4.26bc
Glu	101.71 ± 9.90a	62.96 ± 10.88c	87.21 ± 17.28ab	72.99 ± 0.57bc	79.75 ± 3.08bc
Thr	92.09 ± 3.95a	56.68 ± 10.97b	71.01 ± 20.37b	74.19 ± 2.31ab	68.08 ± 4.67b
Ser	104.98 ± 3.97a	66.40 ± 11.31b	81.53 ± 22.94b	89.15 ± 2.53ab	81.18 ± 5.24b
Gly	71.06 ± 1.78a	44.61 ± 8.00b	54.48 ± 16.82b	57.30 ± 2.04ab	51.20 ± 4.63b
Ala	146.00 ± 6.63a	97.12 ± 17.38c	125.68 ± 21.34ab	117.82 ± 4.02bc	108.91 ± 5.95bc
Pro	70.88 ± 1.38a	40.31 ± 9.23b	52.59 ± 16.27b	50.81 ± 1.32b	51.70 ± 5.71b
Lys	121.57 ± 5.47a	74.10 ± 14.71c	95.78 ± 22.04bc	99.38 ± 3.18ab	88.75 ± 6.11bc
Val	101.79 ± 4.64a	64.34 ± 12.43b	81.94 ± 19.15ab	82.00 ± 2.93ab	75.92 ± 4.74b
Met	27.02 ± 2.06a	18.87 ± 3.57b	22.58 ± 4.61ab	22.97 ± 1.18ab	21.03 ± 0.74b
Ile	87.54 ± 4.29a	56.25 ± 11.03b	70.82 ± 15.34ab	71.06 ± 2.13ab	65.40 ± 4.10b
Leu	141.49 ± 8.44a	97.53 ± 18.06b	120.59 ± 18.90ab	116.76 ± 3.23b	107.54 ± 5.27b
Phe	77.17 ± 6.61a	53.09 ± 9.90b	62.01 ± 13.81ab	61.52 ± 3.37ab	57.13 ± 1.95b
His	44.02 ± 2.13a	24.13 ± 5.26c	30.70 ± 12.27bc	37.26 ± 1.36ab	31.77 ± 2.11bc
Arg	-	-	-	-	-
Tyr	77.40 ± 1.06a	64.67 ± 13.95b	71.24 ± 14.05ab	65.58 ± 3.65ab	58.48 ± 2.93b
Total amino acids (TAA)	1341.16 ± 62.21a	870.22 ± 167.06b	1090.47 ± 246.31ab	1082.16 ± 35.06ab	1005.50 ± 61.46b
Essential amino acid (EAA)	648.67 ± 35.45a	420.85 ± 80.64b	524.72 ± 114.21ab	527.88 ± 18.31ab	483.85 ± 27.56b
Non-essential amino acids (NEAA)	648.47 ± 24.63a	425.24 ± 81.15b	535.05 ± 119.84ab	517.01 ± 15.38b	489.88 ± 31.79b
Conditionally essential amino acids (CEAA)	44.02 ± 2.13a	24.13 ± 5.26c	30.70 ± 12.27bc	37.26 ± 1.36ab	31.77 ± 2.11bc

Different lowercase letters indicate significant differences of 0.05 in the color among treatments.

**Figure 3:**
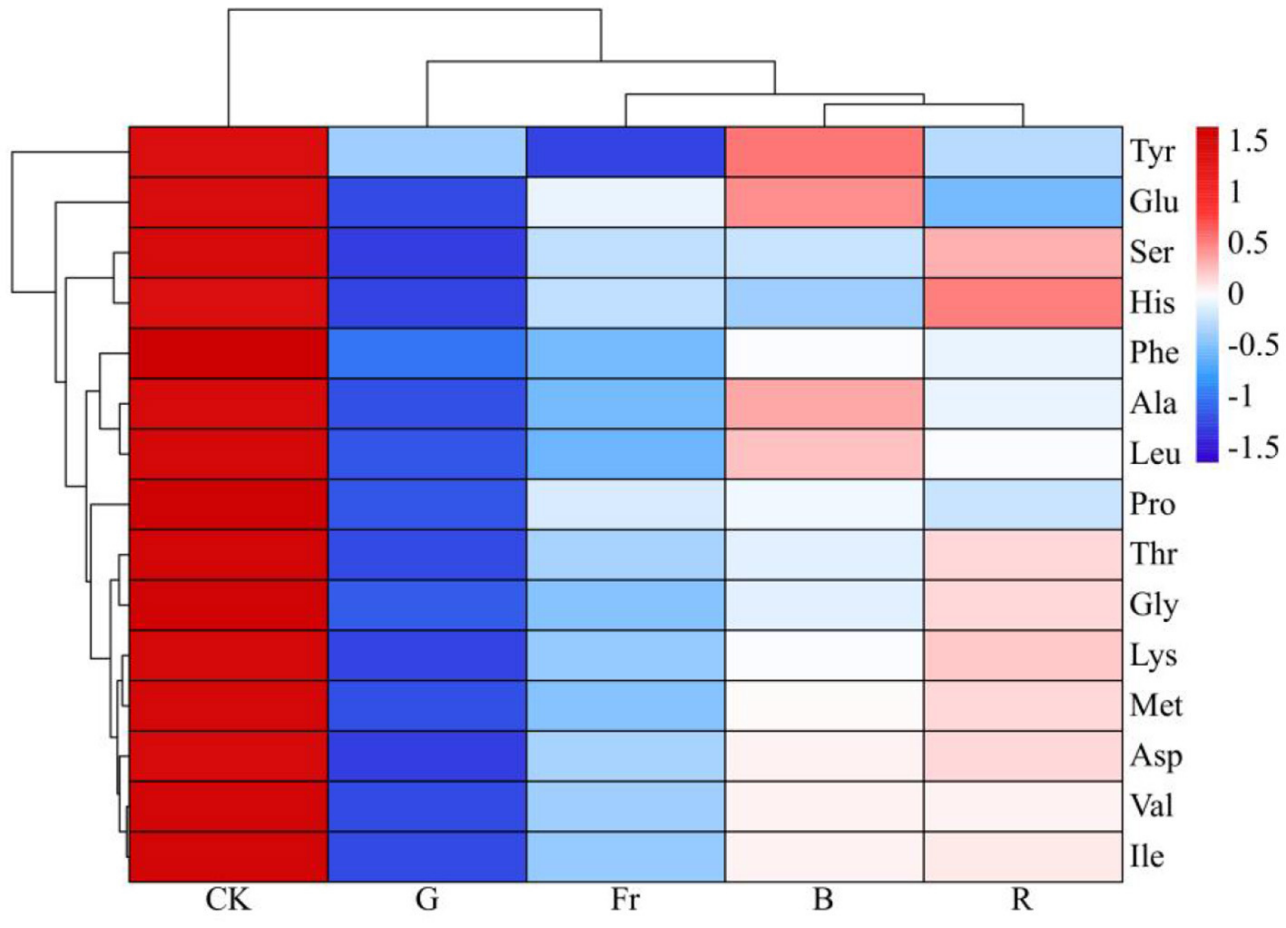
Clustering and heatmap illustration of synthesis of different amino acids under varied LED light treatments.

Among the 15 amino acids identified, threonine (Thr), lysine (Lys), valine (Val), methionine (Met), isoleucine (Ile), leucine (Leu), and phenylalanine (Phe) are essential amino acids (EAA), while asparticacid (Asp), glutamicacid (Glu), serine (Ser), glycine (Gly), alanine (Ala), proline (Pro), and tyrosine (Tyr) are non-essential amino acids (NEAA), moreover histidine (His) is conditionally essential amino acid (CEAA). As shown in Table 3, Figure 3, the contents of total amino acid (TAA), as well as EAA, NEAA and CEAA in *Pleurotus citrinopileatus* exposed to all the treatments were obviously reduced compared to the control, by respectively 18.69%−35.11%, 14.13%−22.88%, 21.20%−52.50% and 38.56%−82.43%. Li et al. (30) reported that Asp and Glu could produce a mushroom flavor. It could be seen in Table 3, except for B treatment, the other light treatments significantly reduced the content of Asp and Glu compared with the control.

The 15 free amino acids identified in the current study included 2 umami amino acids (Asp, Glu), 5 sweet amino acids (Thr, Ser, Gly, Ala, Pro), 7 bitter amino acids (Lys, Val, Met, Ile, Leu, Phe, His), and 1 odorless amino acid (Tyr). As shown in Table 4, although all treatments reduced the content of the flavor amino acids in relative to the control, the proportion of umami and sweet amino acids in the total amino acids [(UAA+SAA)/TAA] was increased while that of bitter amino acids in total amino acids (BAA/TAA) was decreased in mushrooms subjected to B treatment compared with the control, indicating that blue light was more conducive to the synthesis of taste friendly amino acids.

**Table 4:** Content of various odorous amino acids under different LED light treatment.

**Amino acid**	**Amino acid content/(mg/100g)**				
	**CK**	**G**	**B**	**R**	**Fr**
Umami amino acids (UAA)	178.14 ± 9.83a	112.13 ± 21.31c	149.53 ± 28.42ab	136.34 ± 1.86bc	138.42 ± 7.35bc
Sweet amino acids (SAA)	485.01 ± 17.70a	305.12 ± 56.87b	385.29 ± 97.74ab	389.28 ± 12.19ab	361.07 ± 26.19b
Bitter amino acids (BAA)	600.60 ± 33.63a	388.30 ± 74.94b	484.41 ± 106.11ab	490.95 ± 17.37ab	447.54 ± 25.00b
Odorless amino acids (OAA)	77.40 ± 1.06a	64.67 ± 13.95ab	71.24 ± 14.05ab	65.58 ± 3.65ab	58.48 ± 2.93b
UAA/TAA	0.13	0.13	0.14	0.13	0.14
SAA/TAA	0.36	0.35	0.35	0.36	0.36
BAA/TAA	0.45	0.45	0.44	0.45	0.45
(UAA+SAA)/TAA	0.06	0.07	0.07	0.06	0.06

Different lowercase letters indicate significant differences of 0.05 in the color among treatments.

### 3.4 Volatile substances analysis of *Pleurotus citrinopileatus* under different light quality

#### 3.4.1 Qualitative and quantitative analysis of volatile substances

**Figure 4:**
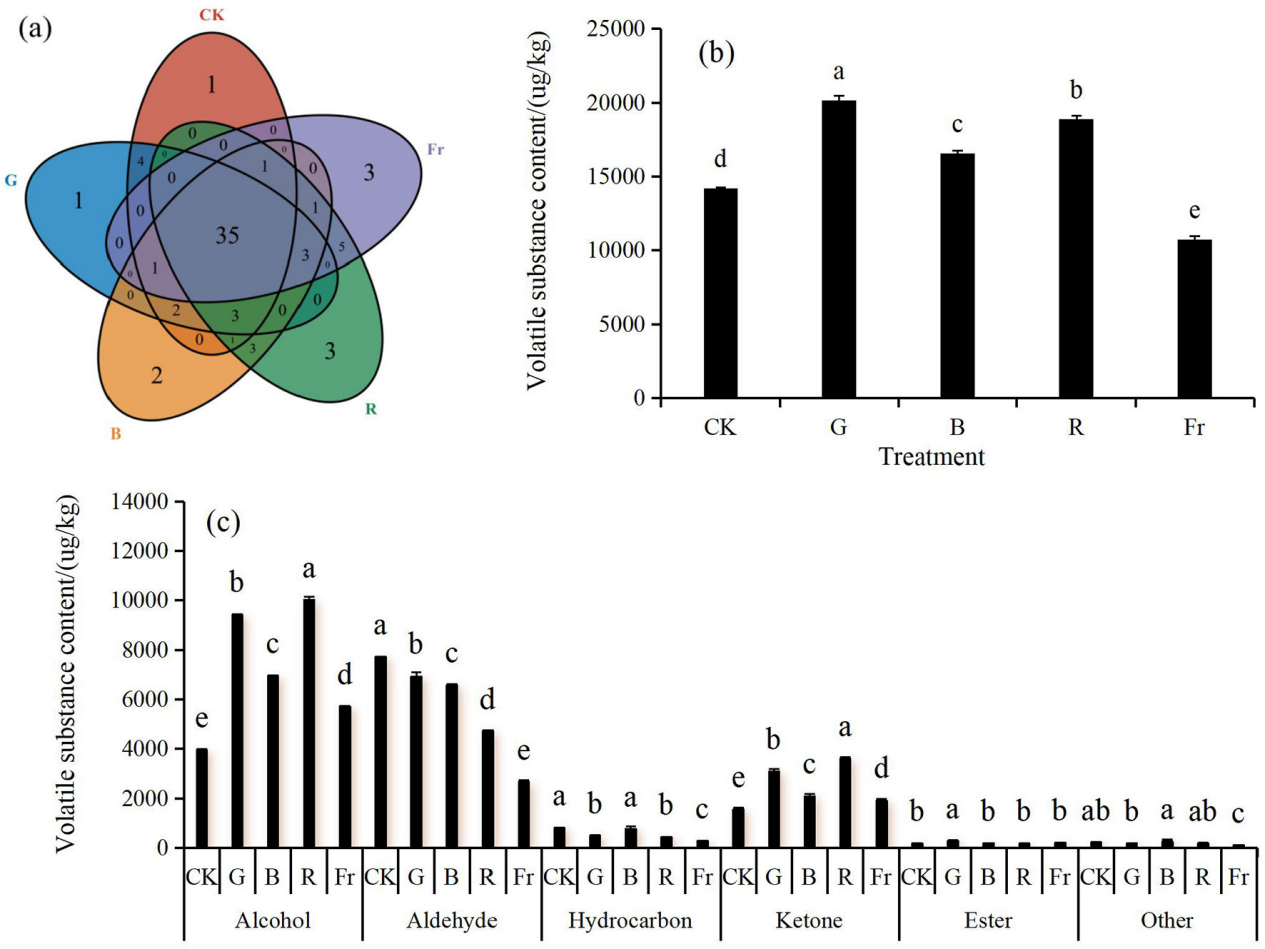
The number of types **(a)**, the content of total volatile substances **(b)** and the content of various types of volatile substances **(c)** under different LED light treatments. Different lowercase letters indicate significant differences between groups (*P* < 0.05).

In terms of the number of volatile substances, totally 69 volatile substances were detected in *Pleurotus citrinopileatus*, including 18 aldehydes, 6 ketones, 24 alcohols, 9 esters, 9 hydrocarbons, and 3 other volatile substances (Table 5), All the light treatments enhanced the number of total volatile substances in comparision to the control, among which, the highest number was detected under R treatment, which was increased by 12.50%. The number of common volatile substances in each treatment was 35 (accounting for 50.72% of total volatile substances), while the specific volatile substances under CK, G, B, R, and Fr treatments were 1, 1, 2, 3, and 3, respectively (Figure 4a).

**Table 5:** Components and contents of volatile substances in *Pleurotus citrinopileatus* under different LED light treatments.

**ID**	**Volatile substances**	**Chemical fomula**	**Chemical abstract service registry number**	**Retention time/min**	**Content/(ug/kg)**				
					**CK**	**G**	**B**	**R**	**Fr**
1	1-Butanol, 3-methyl-	C_5_H_12_O	123-51-3	3.028	50.83 ± 1.27c	261.76 ± 18.8a	78.24 ± 15.08b	25.51 ± 1.90d	25.31 ± 4.12d
2	1-Butanol, 2-methyl-	C_5_H_12_O	137-32-6	3.076	58.07 ± 6.05b	124.56 ± 5.41a	30.96 ± 5.73c	-	14.17 ± 0.68d
3	1-Pentanol	C_5_H_12_O	71-41-0	3.469	30.64 ± 7.01c	54.22 ± 1.98a	57.39 ± 6.91a	43.23 ± 1.75b	15.27 ± 3.98d
	1-Hexanol	C_6_H_14_O	111-27-3	5.226	442.65 ± 18.3b	797.86 ± 20.05a	765.16 ± 45.41a	435.45 ± 22.04b	243.40 ± 11.66c
4	1-Heptanol	C_7_H_16_O	111-70-6	7.287	30.35 ± 8.58d	82.33 ± 2.64a	55.89 ± 4.15b	38.94 ± 1.02c	15.09 ± 1.44e
5	1-Octen-3-ol	C_8_H_16_O	3391-86-4	7.509	798.95 ± 35.09e	1032.94 ± 36.66d	1729.31 ± 60.82	4652.16 ± 26.26a	2658.48 ± 21.14b
6	3-Octanol	C_8_H_18_O	589-98-0	7.838	351.86 ± 1.17e	907.94 ± 6.86c	672.13 ± 4.64d	2762.78 ± 8.94a	1562.26 ± 7.02b
7	2-Octen-1-ol, (Z)-	C_8_H_16_O	26001-58-1	9.290	-	-	-	-	24.02 ± 0.23a
8	1-Octanol	C_8_H_18_O	111-87-5	9.351	200.83 ± 2.94c	302.11 ± 11.76b	293.02 ± 7.49b	440.06 ± 8.70a	202.35 ± 18.98c
9	1,7-Octadien-3-ol, 2,6-dimethyl-	C_10_H_18_O	22460-59-9	9.829	-	-	-	86.70 ± 3.00a	-
10	Phenylethyl Alcohol	C_8_H_10_O	60-12-8	10.278	8.01 ± 1.42cd	63.05 ± 3.28a	26.47 ± 2.00b	11.42 ± 2.34c	6.39 ± 1.40d
11	3-Nonen-1-ol, (E)-	C_9_H_18_O	10339-61-4	10.886	80.61 ± 10.00b	-	629.83 ± 27.43a	87.69 ± 6.54b	55.64 ± 2.85c
12	10-Undecen-1-ol	C_11_H_22_O	112-43-6	10.893	-	161.92 ± 11.9a	-	-	-
13	3-Nonen-1-ol, (Z)-	C_9_H_18_O	10340-23-5	11.033	1148.71 ± 32.23c	4069.17 ± 118.47a	1953.61 ± 100.00b	990.21 ± 60.16d	551.61 ± 18.68e
14	2-Nonen-1-ol, (E)-	C_9_H_18_O	31502-14-4	11.262	28.18 ± 0.01a	33.72 ± 9.54a	-	-	-
15	2-Octen-1-ol, (E)-	C_8_H_16_O	18409-17-1	11.267	-	-	31.84 ± 1.25a	20.20 ± 0.65b	8.47 ± 3.84c
16	1-Nonanol	C_9_H_20_O	143-08-8	11.330	363.43 ± 0.94c	1157.16 ± 11.85a	386.91 ± 13.44b	218.57 ± 15.55d	178.57 ± 10.54e
17	5-Nonanol, 5-methyl-	C_10_H_22_O	33933-78-7	11.434	24.36 ± 1.00a	19.74 ± 5.00b	-	-	-
18	3-Undecanol, 3-ethyl-	C_13_H_28_O	62101-31-9	12.384	283.14 ± 8.19a	212.16 ± 0.76c	224.07 ± 2.89b	189.68 ± 4.27d	119.51 ± 4.98e
19	1-Heptanol, 2-propyl-	C_10_H_22_O	10042-59-8	12.720	14.69 ± 1.97a	9.99 ± 1.77b	13.62 ± 0.50a	-	-
20	5-Decen-1-ol, (Z)-	C_10_H_20_O	51652-47-2	12.929	14.96 ± 1.00a	14.57 ± 2.02a	4.13 ± 1.00b	-	-
21	Z-4-Dodecenol	C_12_H_24_O	40642-37-3	12.981	-	-	-	8.81 ± 0.05a	3.95 ± 0.87b
22	5-Decen-1-ol, (E)-	C_10_H_20_O	56578-18-8	12.977	12.68 ± 2.00a	9.76 ± 1.27b	-	-	-
23	5-Dodecenol	C_12_H_26_O	10203-33-5	13.529	-	-	-	-	14.82 ± 3.56a
24	Pentanal	C_5_H_10_O	110-62-3	2.609	-	-	60.33 ± 10.0a	24.36 ± 0.1b	-
25	Pentanal, 2-methyl-	C_6_H_12_O	123-15-9	3.216	23.39 ± 4.90b	24.30 ± 2.70b	35.58 ± 1.26a	33.65 ± 4.74a	7.17 ± 1.72c
26	Hexanal	C_6_H_12_O	66-25-1	3.962	1990.97 ± 5.80a	922.89 ± 11.71d	1632.25 ± 4.02b	1016.91 ± 3.16c	527.34 ± 24.69e
27	Heptanal	C_7_H_14_O	111-71-7	5.865	151.04 ± 12.38a	85.94 ± 19.74c	116.91 ± 6.26b	65.36 ± 4.63d	34.09 ± 1.48e
28	2-Heptenal, (Z)-	C_7_H_12_O	57266-86-1	7.019	129.62 ± 15.29b	102.40 ± 3.34c	141.60 ± 3.56b	165.20 ± 18.85a	40.21 ± 9.81d
29	Benzaldehyde	C_7_H_6_O	100-52-7	7.179	73.19 ± 12.98a	27.49 ± 1.90bc	34.79 ± 10.73b	27.46 ± 4.79bc	12.74 ± 2.79c
30	Octanal	C_8_H_16_O	124-13-0	7.964	342.64 ± 3.87a	117.60 ± 5.05d	182.78 ± 12.30b	165.41 ± 3.17c	117.47 ± 1084d
31	Benzeneacetaldehyde	C_8_H_8_O	122-78-1	8.860	52.3 ± 4.45c	121.16 ± 0.99b	179.02 ± 7.84a	140.45 ± 16.75b	196.23 ± 24.91a
32	2-Octenal, (E)-	C_8_H_14_O	2548-87-0	9.102	376.73 ± 2.97c	324.40 ± 3.60d	526.59 ± 24.04b	556.23 ± 26.64a	221.27 ± 0.58e
33	Cyclooctanecarboxaldehyde	C_7_H_12_O	2043-61-0	9.873	109.88 ± 4.12b	135.81 ± 2.93a	95.20 ± 3.15c	57.08 ± 2.73d	43.70 ± 1.44e
34	Nonanal	C_9_H_18_O	124-19-6	10.031	1536.75 ± 5.37a	952.71 ± 30.50b	686.4 ± 19.37c	332.60 ± 3.94d	323.76 ± 12.21d
35	2-Nonenal, (E)-	C_9_H_16_O	18829-56-6	11.138	2786.06 ± 32.96b	3844.39 ± 155.37a	2526.19 ± 99.51c	1664.90 ± 1.67d	965.02 ± 94.82e
36	trans-Undec-4-enal	C_11_H_20_O	68820-35-9	11.767	-	25.14 ± 3.00d	69.87 ± 2.77a	43.19 ± 1.38c	59.11 ± 4.01b
37	cis-4-Decenal	C_10_H_18_O	21662-09-9	11.799	86.14 ± 1.02ab	84.44 ± 4.11b	84.59 ± 2.13b	90.08 ± 2.91a	-
38	Decanal	C_10_H_20_O	112-31-2	11.984	10.14 ± 2.00a	-	4.74 ± 0.19c	6.54 ± 0.21b	-
39	2,4-Dodecadienal, (E,E)-	C_12_H_20_O	21662-16-8	13.626	-	41.82 ± 7.21b	80.72 ± 8.48a	90.98 ± 10.17a	25.65 ± 8.78c
40	2,4-Decadienal, (E,E)-	C_10_H_16_O	25152-84-5	14.055	53.73 ± 0.70c	116.53 ± 13.71b	121.62 ± 2.15b	171.67 ± 3.88a	57.89 ± 19.37c
					**CK**	**G**	**B**	**R**	**Fr**
41	2-Undecenal	C_11_H_20_O	2463-77-6	14.819	-	-	-	41.67 ± 1.80a	18.10 ± 3.96b
42	1-Octene	C_8_H_16_	111-66-0	3.798	-	-	-	13.01 ± 2.19a	-
43	Nonane	C_9_H_20_	111-84-2	5.805	-	-	-	8.01 ± 0.02a	7.71 ± 0.27b
44	6-Tridecene, (E)-	C_13_H_26_	6434-76-0	8.497	208.55 ± 2.04a	117.84 ± 17.55b	119.97 ± 0.77b	107.26 ± 0.77b	75.45 ± 3.64c
45	1,3-Hexadiene, 3-ethyl-2-methyl-	C_9_H_16_	61142-36-7	8.570	113.81 ± 6.74b	68.36 ± 9.05d	184.79 ± 4.08a	93.07 ± 9.48c	34.01 ± 5.10e
46	5-Tridecene, (Z)-	C_13_H_26_	25524-42-9	9.827	230.36 ± 9.15a	114.96 ± 7.89b	108.12 ± 1.00b	-	55.72 ± 0.48c
47	Cyclohexane, 1-methyl-2-propyl-	C_10_H_20_	4291-79-6	10.650	-	-	14.38 ± 2.68a	-	-
48	Cyclohexene, 3-methyl-6-(1-methylethyl)-	C_10_H_18_	5256-65-5	11.668	-	-	42.57 ± 2.45a	-	-
49	Cyclohexene, 3,3,5-trimethyl-	C_9_H_16_	503-45-7	13.701	42.64 ± 1.29a	-	-	-	-
50	2,4-Heptadiene, 2,4-dimethyl-	C_9_H_16_	74421-05-9	13.704	-	-	54.08 ± 2.60a	15.84 ± 0.72b	-
51	2-Propen-1-amine	C_3_H_7_N	107-11-9	3.092	-	-	-	8.79 ± 0.53a	-
52	2-Amino-5-methylbenzoic acid	C_8_H_9_NO_2_	2941-78-8	5.944	18.41 ± 3.27b	27.50 ± 2.17ab	39.99 ± 15.32a	27.18 ± 0.84ab	36.97 ± 10.90a
53	Furan, 2-pentyl-	C_9_H_14_O	3777-69-3	7.696	194.18 ± 9.32ab	152.99 ± 6.67b	211.89 ± 11.49a	155.04 ± 10.51b	74.12 ± 11.60c
54	1-Octen-3-one	C_8_H_14_O	4312-99-6	7.423	62.89 ± 4.82e	122.14 ± 9.90c	253.15 ± 3.81b	185.12 ± 11.89a	7.74 ± 3.40d
55	3-Octanone	C_8_H_16_O	106-68-3	7.593	1347.54 ± 44.12d	2846.95 ± 73.16b	1718.97 ± 71.23c	3187.27 ± 32.70a	1799.35 ± 80.41c
56	2-Pentylcyclopentanone	C_10_H_18_O	4819-67-4	9.054	61.15 ± 10.85a	48.80 ± 10.06a	47.25 ± 11.04a	23.85 ± 0.76b	23.43 ± 3.23b
57	2-Acetonylcyclopentanone	C_7_H_10_O_2_	1670-46-8	9.267	51.31 ± 9.10ab	29.65 ± 2.80c	44.15 ± 3.46b	55.63 ± 7.63a	-
58	5-Decanone, 2-methyl-	C_11_H_22_O	54410-89-8	12.729	-	-	-	6.45 ± 0.30a	4.57 ± 0.28b
59	2-Dodecanone	C_12_H_24_O	6175-49-1	13.539	37.48 ± 6.65a	24.17 ± 4.85b	21.04 ± 5.29b	33.29 ± 3.78a	-
60	2-Propenoic acid, 2-methyl-, ethenyl ester	C_6_H_8_O_2_	4245-37-8	2.602	-	-	-	-	69.28 ± 4.39a
61	Heptanoic acid, methyl ester	C_8_H_16_O_2_	106-73-0	8.357	-	-	13.35 ± 1.50a	6.30 ± 0.99b	-
62	Octanoic acid, methyl ester	C_9_H_18_O_2_	111-11-5	10.368	9.94 ± 1.77b	14.66 ± 3.84a	10.55 ± 2.03b	10.71 ± 0.29b	4.08 ± 0.90c
63	3-Nonenoic acid, methyl ester	C_10_H_18_O_2_	13481-87-3	12.205	85.16 ± 11.47b	126.67 ± 3.98a	89.47 ± 3.16b	71.69 ± 1.14c	49.44 ± 4.80d
64	Nonanoic acid, methyl ester	C_10_H_20_O_2_	1731-84-6	12.272	28.76 ± 0.37b	61.00 ± 1.58a	29.48 ± 6.11b	19.91 ± 1.01c	13.77 ± 0.34d
65	2(3H)-Furanone, dihydro-5-pentyl-	C_9_H_16_O_2_	104-61-0	14.777	34.23 ± 4.18a	36.22 ± 17.33a	-	-	-
66	Dimethyl phthalate	C_10_H_10_O_4_	131-11-3	16.249	-	9.15 ± 1.00b	15.07 ± 3.00a	19.22 ± 5.07a	17.28 ± 3.78a
67	Pentadecanoic acid, methyl ester	C_16_H_32_O_2_	7132-64-1	21.752	-	-	-	6.65 ± 1.61a	7.03 ± 1.92a
68	Hexadecanoic acid, methyl ester	C_17_H_34_O_2_	112-39-0	23.573	16.15 ± 2.49b	36.15 ± 2.59a	32.86 ± 6.91a	39.36 ± 7.72a	37.74 ± 7.82a

“–” indicated that the volatile substance was not detected.

Different lowercase letters indicate significant differences of 0.05 in the color among treatments.

In terms of the content of total volatile substance, G, B, and R treatments significantly increased the total volatile substance content by 42.03%, 16.71%, and 33.01% compared to the control, while that under Fr treatment significantly decreased by 24.44% (*P* < 0.05) (Figure 4b). As regards of a certain type of volatile substance, monochromatic light irradiation (G, B, R, or Fr) significantly increased the content of alcohols and ketones by 44.54%−138.78% and 22.03%−130.18% (*P* < 0.05) compared to the control, among which, the highest contents were both detected under R treatment. On the contrary, monochromatic light irradiation decreased the content of aldehydes and hydrocarbons by 10.30%−65.69% and 3.98%−64.85%, respectively. This might indicate that monochromatic light was more conducive to the synthesis of alcohols and ketones, but not for the synthesis of aldehydes and hydrocarbons (Figure 4c).

#### 3.4.2 OAV analysis of key volatile substances

**Figure 5:**
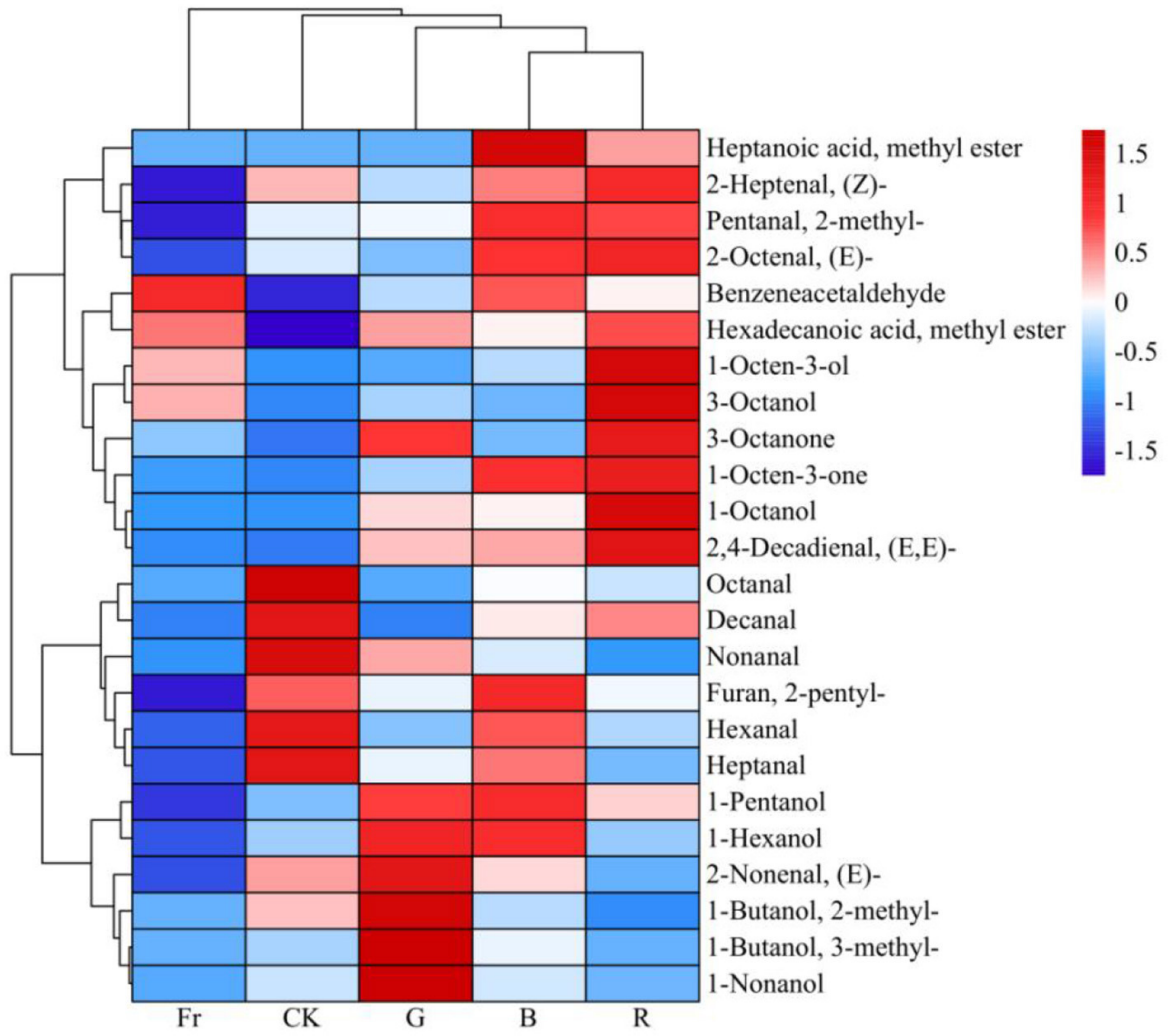
OAV values of volatile substances under different LED light treatments.

Twenty four key volatile substances of *Pleurotus citrinipileatus* were detected in the present study, including 8 alcohols, 11 aldehydes, 2 ketones, 2 esters, and 1 other (Figure 5). Alcohols especially octanols were characteristic substances commonly existed in edible fungi. Three octanols with OAV >1 were detected in the present study, among which, 1-octen-3-ol was closely related to the “mushroom aroma”. As shown in Figure 5, the highest OAV value of 1-octen-3-ol was detected in *Pleurotus citrinipileatus* exposed to R treatment. Low odor threshold of aldehydes resulted in a strong odor of mushrooms. 11 aldehydes were detected in this study, among which, 2-nonenal, (E)- in *Pleurotus citrinipileatus* subjected to G treatment presented the highest OAV value, resulting in a sweet aroma. Ketones endowed edible fungi with floral and fruity aromas, and the fragrance was long-lasting. Two key ketones that were 1-octen-3-one and 3-octanone were detected in *Pleurotus citrinipileatus* under all the treatments, both of which presented the highest OAV value under R treatment. Esters could contribute to the pleasant fruit aroma of *Pleurotus citrinipileatus*. The highest OAV value of heptanoic acid, methyl ester was detected in *Pleurotus citrinipileatus* exposed to B treatment. In addition, the highest OAV values of 1-pentanol, furan, and 2-pentyl- were all detected under B treatment (31).

### 3.5 OPLS-DA analysis

**Figure 6:**
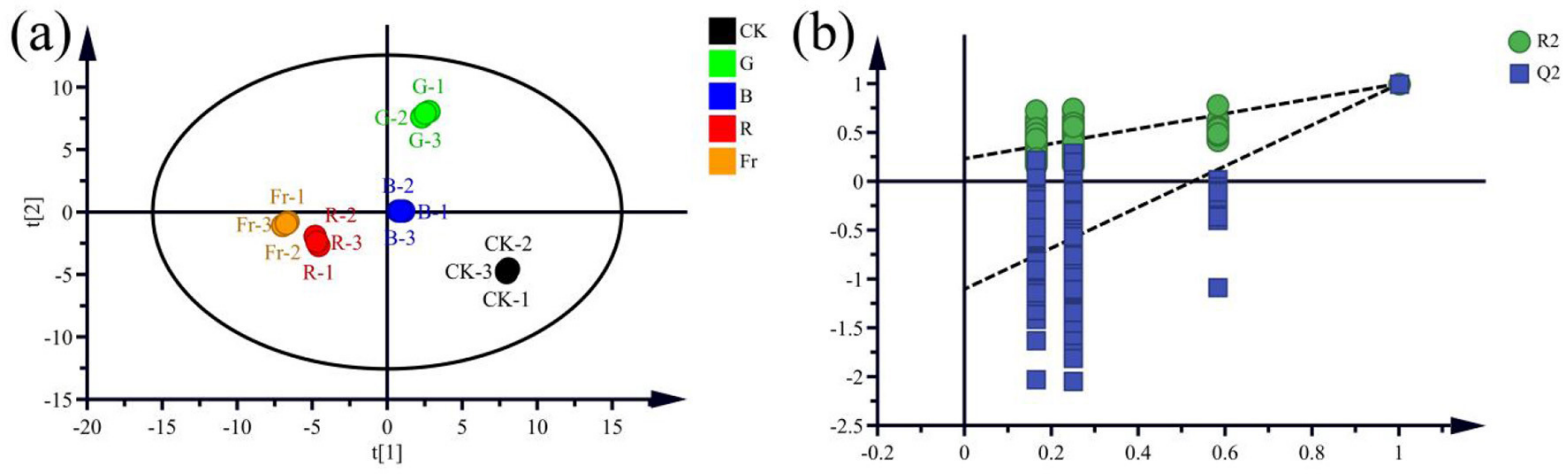
OPLS-DA **(a)** and cross-validation model **(b)** of free amino acids and volatile substances in *Pleurotus citrinopileatus*.

OPLS-DA analysis was conducted with 15 free amino acids and 69 volatile substances as dependent variables and different light treatments as independent variables. The fitting index of the independent variable (${\text{R}}_{\text{x}}^{2}$), the dependent variable (${\text{R}}_{\text{y}}^{2}$), and the model prediction index (Q^2^) in this analysis were 0.978, 0.997, and 0.991, respectively. As shown in Figure 6a, the data points within the group were concentrated, while the data points among groups were scattered, indicating that the experimental methods and instruments were reliable and stable, and there were significant differences among groups. Therefore, the content of free amino acids and volatile substances could be used for sensitivity analysis of *Pleurotus citrinipileatus* to different light qualities. 200 permutation tests showed that the intersection point between the Q^2^ regression line and the vertical axis was < 0, indicating that the model did not have overfitting and the model validation was effective (Figure 6b).

## 4 Discussion

Light is one of the most important environmental signals that affect the growth of edible fungi, regulating their growth and physiological processes through photoreceptors.

Our experimental observations revealed distinct photomorphogenic abnormalities in *Pleurotus citrinopileatus* under red and far-red irradiance, characterized by three key phenotypic alterations: soft stipe, thin pileus, and attenuated carotenoid deposition. It has been shown that the synthesis of membrane transporters which sensed external stimuli and maintained biological activity in edible fungal was ineffective under red or far-red light irradiation (21). This might explain the phenomenon of deformities in *Pleurotus citrinopileatus* exposed to R or Fr treatments in the current study. The growth of *Pleurotus citrinopileatus* under R and Fr treatments appeared normal in the early stage (button stage and young mushroom stage), which might be due to the accumulated membrane transporters during the hyphal stage. However, with the irradiation of red or far-red light during the fruiting body stage, the synthesis of membrane transporters were inhibited until insufficient to maintain the normal growth of *Pleurotus citrinopileatus*.

Blue light irradiation made significant morphometric alterations in *Pleurotus citrinopileatus*, manifesting as a 12.52% reduction in stipe length, contrasted by 35.52% and 18.30% increases in stipe diameter and pileus diameter, respectively, compared with the control (*P* < 0.05). At the same time, B treatment significant increased the weight of fruiting bodies by 23.66% compared to the control (*P* < 0.05). These were similar with Jang et al.'s (32) and Kamada et al.'s (33) findings that the pileus diameter of *Agaricus bisporus* and *Coprinopsis cinerea* were larger under blue light irradiation compared with green or red light. It might be due to that the blue light receptors (*WC-1 and WC-2*) mainly related to the development of the fruiting bodies presented the highest level under blue light irradiation (34). Besides, it was reported that the activity of extracellular enzymes such as cellulase, peroxidase, and laccase was up-regulated by blue light in relative to red light or white light, thus accelerating the decomposition of the cultivation materials which provided nutrients for fruiting body development (18, 35). This might also accounted for the best growth of *Pleurotus citrinopileatus* exposed to B treatment in the present study.

The color of mushroom reflects its freshness and affects consumers' willingness (36). Our study found that the color of pileus was more vibrant under B treatment, with higher. color spectral parameters of C and Hue. Zhang et al. (37) has reported that the color of edible fungi was controlled by pigments such as carotenoids which was related to the blue light signaling pathway and was synergistically regulated by blue light photoreceptors This might partially explain the findings that *Pleurotus citrinopileatus* displayed well color under B treatment in this study. It implied that blue light was beneficial for *Pleurotus citrinopileatus* cultivation from the perspective of coloring.

Free amino acids and volatile substances are the main substances that determine the taste and aroma of *Pleurotus citrinopileatus*. In the current study, blue light irradiation increased the proportion of umami and sweet amino acids in total amino acids (UAA+SAA)/TAA, while decreased that of bitter amino acids in total amino acids (BAA/TAA) in *Pleurotus citrinopileatus*. This might be due to that blue light irradiation tended to induce the conversion of carbohydrates into umami and sweet amino acids, rather than bitter amino acids (38).

Volatile substances such as aldehydes, ketones, and alcohols were converted from flavor precursors through histidine metabolism, glutathione metabolism, and unsaturated fatty acid metabolism under the action of a series of flavor synthases. Edible fungi sensed different light qualities through photoreceptors, which affected the synthesis of precursors and the activity of synthases, thereby affecting the content of volatile substances. It has been shown that monochromatic light enhanced the synthesis of substances such as carotenoids, fatty acids, phenylalanine and branched chain amino acids, which played vital roles during the synthesis of volatile substance (39). Similar results were observed in the present study, in which, the number and content of total volatile substances were increased by G, B, and R treatments compared with the control. It was reported that blue light irradiation increased enzyme activity in edible fungi. Therefore, this study found that B increased the OAV of key aroma compounds might be due to the increased activity of synthetic enzymes by blue light, greatly increasing the content of these substances and making them characteristic flavor compounds. Feng et al. (24) also reported that most aroma compounds in dried *Suillus granulatus* displayed higher OAV value under blue light than other light qualities such as green, red, yellow or white light. It implied that blue light was beneficial for the mushroom flavor. In addition, the current study showed that *Pleurotus citrinopileatus* displayed higher OAV value of 1-octen-3-ol under R treatment than B, while the opposite results were detected in the study of Feng et al. (24). The inconsistence may be attributed to the genotypic difference between the two mushroom species or the physiology metabolic differences between pre-harvest and post-harvest mushrooms.

Blue light was the recommended light quality in this study due to the best overall performance in terms of growth, coloring, taste and aroma. However, it was worth mentioning that although mushrooms could not grow normally under R, R increased the OAV values of certain key ketones (e.g., 1-octen-3-one and 3-octanone) in *Pleurotus citrinipileatus*, giving them a strong mushroom flavor. Thus, it may be possible to try applying red light during the post-harvest stage of *Pleurotus citrinipileatus*.

## 5 Conclusion

Monochromatic red light or far-red light caused abnormal appearance in *Pleurotus citrinopileatus*, showing soft stipe, thin pileus and shallow color, while monochromatic blue light was proved to be beneficial for the growth and coloring of *Pleurotus citrinopileatus*. Compared with the control, the stipe diameter, pileus diameter, and fruiting body weight significantly increased by 35.52%, 18.30%, and 23.66%, respectively (*P* < 0.05). The color of *Pleurotus citrinopileatus* was more plump under blue light treatment, among which the spectral color parameters C and Hue increased by 2.72% and 1.64%, respectively. Moreover, blue light irradiation increased the proportion of umami and sweet amino acids while decreased that of bitter amino acids in total amino acids, as well as increased the odor activity value (OAV) of key aroma compounds, thereby making mushrooms present better taste and aroma.

## Data Availability

The original contributions presented in the study are included in the article/supplementary material, further inquiries can be directed to the corresponding authors.
